# Dietary-Based Diabetes Risk Score and breast cancer: a prospective evaluation in the SUN project

**DOI:** 10.1007/s13105-024-01036-9

**Published:** 2024-09-05

**Authors:** Inmaculada Aguilera-Buenosvinos, Miguel A. Martínez-González, Andrea Romanos-Nanclares, Rodrigo Sánchez-Bayona, Carlos E. de Andrea, Ligia J. Domínguez, Estefania Toledo

**Affiliations:** 1 Universidad de Navarra Departamento de Medicina Preventiva y Salud Pública, Pamplona, 31008 Spain; 2 IdiSNA, Navarra Institute for Health Research, Pamplona, 31008 Spain; 3 Centro de Investigación Biomédica en Red Área de Fisiología de la Obesidad y la Nutrición (CIBEROBN), Madrid, 28029 Spain; 4 Department of Nutrition, Harvard T. H. Chan School of Public Health, Harvard University, Boston, MA 02115 USA; 5 Channing Division of Network Medicine, Department of Medicine, Brigham and Women’s Hospital, Harvard Medical School, Boston, MA USA; 6 Medical Oncology Department, Hospital Universitario 12 de Octubre, Madrid, 28041 Spain; 7 Department of Pathology, Clinica Universidad de Navarra, Pamplona, Navarra 31008 Spain; 8 Centro de Investigación Biomédica en Red, Área de Cáncer, Madrid, 28029 Spain; 9 Geriatric Unit, Department of Internal Medicine and Geriatrics, University of Palermo, Palermo, Italy; 10 Faculty of Medicine and Surgery, University of Enna “Kore”, Enna, Italy

**Keywords:** Breast cancer, Dietary-based diabetes risk score, Diet, Prospective, Cohort

## Abstract

An association between type 2 diabetes (T2D) and breast cancer risk has been reported. This association can be potentially explained by alteration of the insulin/IGF system. Therefore, we aimed to prospectively investigate whether a previously reported Dietary-Based Diabetes Risk Score (DDS) inversely associated with T2D was also associated with breast cancer risk in the SUN (“Seguimiento Universidad de Navarra”) cohort. We followed up 10,810 women (mean age = 35 years, SD = 11 years) for an average of 12.5 years during which 147 new cases of invasive breast cancer were diagnosed. A validated 136-item FFQ was administered at baseline and after 10 years of follow-up. The DDS (range: 11 to 55 points) positively weighted vegetables, fruit, whole cereals, nuts, coffee, low-fat dairy, fiber, PUFA; while it negatively weighted red meat, processed meats, and sugar-sweetened beverages. The DDS was categorized into tertiles. Self-reported medically diagnosed breast cancer cases were confirmed through medical records. We found a significant inverse association between the intermediate tertile of the DDS score and overall breast cancer risk (Hazard ratio, HR_T2 vs. T1_= 0.55; 95% CI: 0.36–0.82) and premenopausal breast cancer risk (HR_T2_= 0.26; 95% CI: 0.13–0.53), but not for the highest tertile. This association was stronger among women with a BMI < 25 kg/m^2^ (p_interaction_: 0.029). In conclusion, moderate adherence to the DDS score was associated with a lower risk of breast cancer, especially among premenopausal women and women with a lower BMI. These findings underscore the importance of antidiabetic diet in reducing the risk of breast cancer.

**Supplementary Information:**

The online version contains supplementary material available at 10.1007/s13105-024-01036-9.

## Key Points

Moderate adherence to the DDS was associated with a lower risk of breast cancer.

The observed inverse association was somewhat stronger among lean women.

BMI may have a differentially impact between T2D and breast cancer.

**Supplementary Information:**

The online version contains supplementary material available at 10.1007/s13105-024-01036-9.

## Introduction

Breast cancer is the leading malignancy in females worldwide. In 2020, 2.3 million new diagnoses of breast cancer were made in women worldwide and it was the first cause of cancer death among women, according to Globocan [1]. Breast cancer is associated with various modifiable risk factors –such as alcohol, tobacco consumption, or obesity [2, 3], and non-modifiable risk factors –such as inherited gene mutation (e.g., BRCA 1 and /2, BRCA1 and 2 DNA repair associated), age, and family history of breast cancer [4, 5].

Type 2 diabetes (T2D) is associated with an increased risk of several types of cancer, including breast cancer [6, 7]. Specifically, women with diabetes are at greater risk of developing triple-negative breast cancer (TNBC), compared to women without diabetes [8]. Furthermore, T2D is also associated with increased risk for postmenopausal breast cancer [9]. Moreover, metabolic syndrome has been suggested to be a risk and prognostic factor for many cancers, including postmenopausal breast cancer [10].

Wolf et al. suggested three mechanisms linking T2D with breast cancer: activation of the insulin pathway, activation of the insulin-like-growth-factor (IGF) pathway, and regulation of endogenous sex hormones caused by increased production of oestradiol and androgens combined with decreased liver production of sex hormone binding globulin (SHBG), [11, 12]. Also, chronic hyperglycemia may be a mediator in the association between diabetes and breast cancer risk [13, 14]. Many of the abovementioned factors can be modulated through a healthy lifestyle, and, more specifically, through diet. In fact, a healthy lifestyle and efficient management of chronic metabolic diseases prevent the risk of developing diabetes on the one hand and breast cancer on the other hand, as well as other chronic diseases [15, 16]. Therefore, encouraging lifestyle modifications to reduce the risk of developing insulin resistance and hyperinsulinemia may be a possible strategy for breast cancer primary prevention in addition to contributing to the reduction of other comorbidities.

Recently, an umbrella review [17] summarized the effect of dietary factors and diet interventions on T2D risk. It was concluded that healthy dietary patterns such as the Mediterranean diet and the Dietary Approaches to Stop Hypertension (DASH) diet, as well as a high consumption of whole grains, low-fat dairy products, yogurt, olive oil, chocolate, fiber, magnesium, and flavonoids significantly reduced the risk of T2D. Interestingly, many of these dietary factors are related to breast cancer prevention as well [18]. On the contrary, dietary factors such as foods high in glycemic index (GI) – including sugar-sweetened beverages (SSBs) and refined grains–, red meat, and saturated and *trans* fats have been associated with an increased risk of T2D [17] and they have also been associated with an increased risk of breast cancer [18].

In this context, the Dietary-Based Diabetes Risk Score (DDS), which addresses an optimal dietary pattern for diabetes prevention incorporating several of the abovementioned foods and nutrients [19], was strongly inversely associated with the risk of T2D. However, to the best of our knowledge, no study so far has prospectively related this score to breast cancer risk. Given that 10–20% of patients with breast cancer have T2D [9], proposing this research would be justified from the prevention point of view of two highly prevalent diseases responsible for a high disease burden. Therefore, we aimed to investigate the association between the DDS score and breast cancer incidence within the framework of the SUN Project.

## Materials and methods

### Study sample

**Fig. 1:**
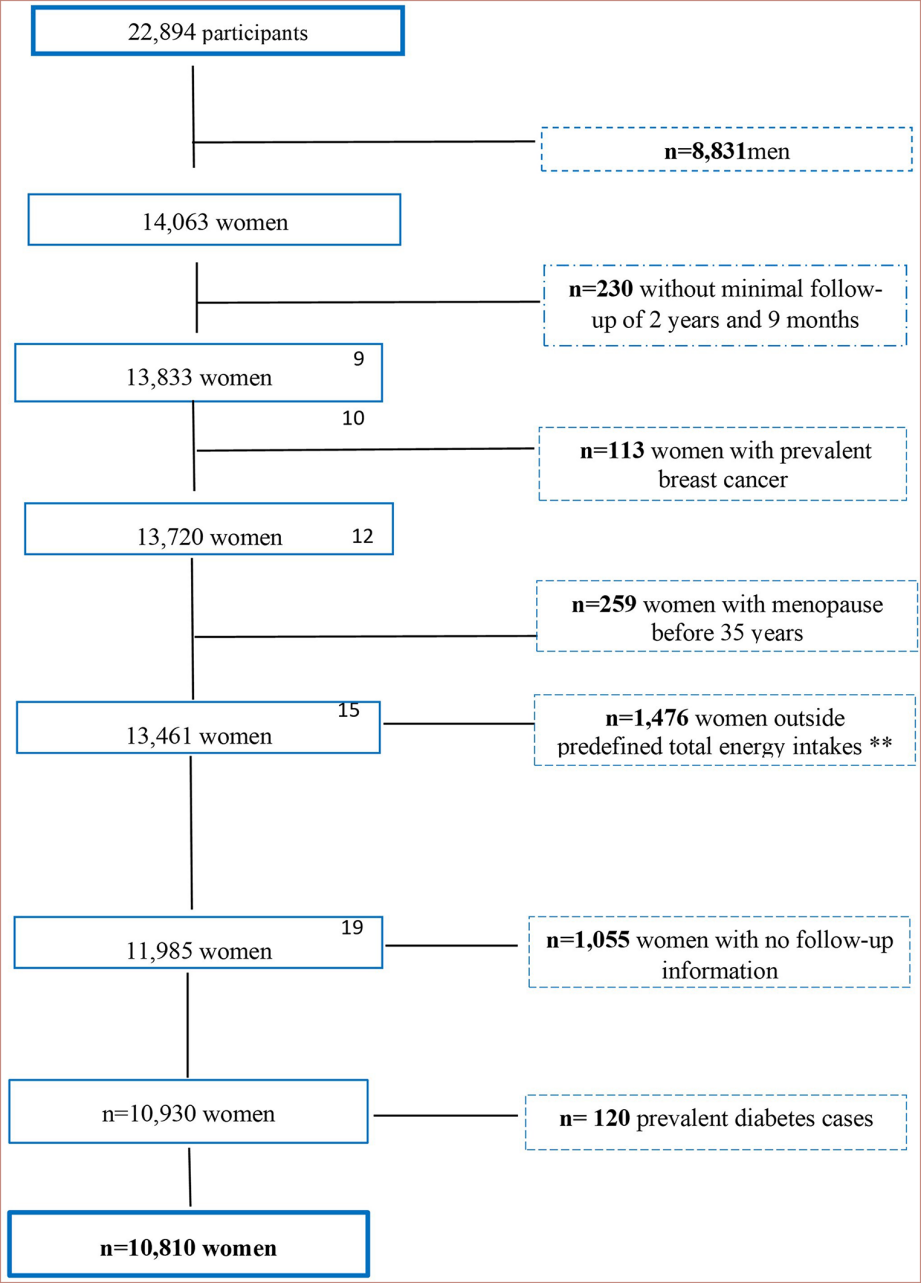
Flowchart representing the inclusion and exclusion criteria for the selection of participants of the SUN Project (“Seguimiento Universidad de Navarra”) included in this analysis. SUN Project, 1999–2019. * To ensure a 2 year and 9-months follow-up. **Energy limits proposed by Willett (2013) [22]: 500–3500 kcal/day

The “Seguimiento Universidad de Navarra” (SUN) Project is a prospective, multipurpose cohort of Spanish university graduates. The study methods have been described in more detail elsewhere [20]. Briefly, the SUN project is a dynamic cohort where the recruitment is permanently open aiming to assess the relationship between diet and chronic diseases. It was developed inspired by the models of the Nurses’ Health Study and the Health Professionals Follow-Up Study. Recruitment started in December 1999. Participants are middle-aged university graduates from different Spanish regions. After recruitment, follow-up questionnaires are sent every 2 years to update information on diet and lifestyle and collect information on health outcomes, which might have happened in the previous 2 years. For participants lost during the follow-up, the National Death Index is annually consulted to assess their vital status and their eventual cause of death. By 2019, 22,894 participants were recruited. Considering the exclusion of 8,831 men, and to ensure a follow-up time of at least 2 years, we included only those women who were recruited before March 2017. Out of 13,833 eligible women, we excluded 113 participants with self-reported history of breast cancer at baseline, 259 women who reported menopause before 35 years, 1476 women with total energy intake outside of predefined limits [21], 1,055 women with no follow-up information and 120 women with diabetes at baseline. Our final sample for the present analysis of DDS and incidence of breast cancer included 10,810 women (Fig. 1).

The present study is in line with the guidelines stated in the Declaration of Helsinki, and all procedures involving participants were approved by the Ethics Committee of the University of Navarra (30 August 2001). Voluntarily given informed consent (protocol code 010830) through free fulfilment of the baseline questionnaire was gathered from all participants according to the methods approved by our Ethics Committee.

### Dietary assessment and DDS

The baseline questionnaire included a previously validated 136-item food-frequency questionnaire (FFQ) [23, 24]. The reproducibility of the FFQ has been previously assessed [25, 26]. The FFQ assessed habitual food consumption over the previous 12 months and included nine categories of response for the frequency of consumption, ranging from ‘never/seldom’ to ‘more than six times per day’. Diet was assessed at baseline and after 10-years to reduce measurement error and within-person variation in the assessment of long-term intake.

Based on a previously developed DDS [19], we prospectively evaluated the association between the abovementioned score and breast cancer risk within the SUN cohort study during the follow-up. Supplemental etable 1 shows the scoring criteria for the foods and nutrients included in the DDS. To calculate the DDS, we first divided the 11 dietary groups into 8 positively weighted groups (vegetables, fruits, total dietary fiber, whole-grain cereals, nuts, coffee, polyunsaturated fatty acids, and low-fat dairy products) and 3 negatively weighted groups (red meat, processed meat, and sugar-sweetened beverages and fruit juices) based on their association with breast cancer risk. Each dietary group was assigned a score ranging from 1 to 5 points according to quintiles of intake, with a higher score indicating a healthier dietary pattern. The scores for each dietary group were added up to obtain the total score. Subsequently, the scores were divided into three tertiles for analysis, allowing for a more detailed examination of the relationship between dietary patterns and breast cancer risk.

To build the DDS, we considered the consumption (g/d) of eight nutritional exposures which have shown inverse associations with T2D (vegetables, fruit, fiber, whole-grain cereals, nuts, coffee, polyunsaturated fatty acids [PUFA], and low-fat dairy products), and three food groups which have shown direct associations with an increased incidence of T2D (red meat, processed meat, and sugar-sweetened beverages and fruit juices [SSB]). We did not consider alcohol consumption from the original score because of its proven harmful relationship with breast cancer, but it was used as a confounder in the multivariable adjusted model. We adjusted the consumption of each nutritional variable for total energy intake with the residual method [27]. The energy-adjusted estimates (residuals) were ranked according to quintiles (assigning a value of 1 for the first quintile, 2 for the second quintile, and successively until the value of 5 was assigned to the fifth quintile). The quintiles for the food groups which have shown increased risk of incident T2D were reversely scored.

Finally, the DDS was calculated as the sum of these quintiles with a range from 11 (lowest adherence) to 55 (highest adherence) points. Adherence to the DDS was subsequently classified into roughly tertiles: cut-off points 13–30 for T1, 31–36 for T2 and 37–54 for T3. A higher score indicates a healthier overall diet and a decreased risk of T2D. Since diet may change over time, we considered cumulatively averaged DDS after 10-years of follow-up using time-dependent Cox models with changing cumulative exposures throughout the follow-up period.

### Ascertainment of incident breast cancer cases

For the present analysis, incidence of breast cancer was the primary and unique endpoint. Participants were asked whether they had received a medical diagnosis of breast cancer at baseline and during follow-up. Participants who reported a breast cancer medical diagnosis were asked for a copy of their medical records. Then, a trained oncologist -blinded with respect to dietary exposures- confirmed the cases. If the participant was lost during the follow-up or had an unidentified cause of death, the National Death Index was consulted each year to identify deceased cohort members and, in case they had passed away, confirm the cause of death. Both cases were adjudicated by a trained oncologist and deaths due to breast cancer were considered as confirmed breast cancer cases (*n* = 147). Finally, our analysis included 10,810 women during the follow-up period (median follow-up = 12.5 years).

### Assessment of covariates

The baseline questionnaire also included information about participants’ socio-demographic characteristics, medical history (prevalent chronic diseases), health-related habits, lifestyles, anthropometric data, physical activity, and family history of breast cancer [28, 29].

Covariates included: height, years at university, family history of breast cancer (none, after 45 years, or before 45 years), smoking status (never smoker, former smoker, current smoker), lifetime tobacco exposure (pack-years), physical activity (METs-h/week), TV watching (h/day), alcohol intake (g/day, continuous), BMI (< 25, 25–<30, ≥ 30 kg/m^2^), age at menarche (< 10 years, 10–11 years, 12–13 years, ≥ 14 years), age at menopause (< 50 years, ≥ 50 years), history of pregnancy (age < 25 years and nulliparous, age ≥ 25 years and nulliparous, first pregnancy before the age of 30 years, first pregnancy being 30 years old or older), months of breastfeeding (continuous), use of hormone replacement therapy (yes/no), energy intake (kcal/day), prevalence of diabetes, family history of diabetes, and use of oral contraceptives (yes/no). Cumulative averages for all dietary variables.

### Statistical analysis

Baseline characteristics of participants are described as the means and standard deviations according to tertiles of adherence to the DDS (Table 1) whose components were adjusted for total energy intake using the residual method [21]. For the main analysis, participants were divided into tertiles according to their adherence to the DDS. We also presented the baseline characteristics of participants adjusted for age with the inverse probability weighting (IPW) method (Supplemental eTable 2).

**Table 1:** Baseline characteristics of participants according to categories of the Dietary-Based Diabetes Risk Score : the Seguimiento Universidad De Navarra (SUN) cohort: 1999–2019

	Tertiles of the Dietary-Based Diabetes Risk Score		
Variable	Tertile 1	Tertile 2	Tertile 3
**N**	3,805	3,781	3,224
**Median DDS (range)**	27 (13–30)	33 (31–36)	40 (37–54)
**Age (years)**	31.9 (8.9)	35.4 (10.4)	38.6 (11.2)
**Body mass index (kg/m** ^**2**^ **) (%)**	21.8 (3.0)	22.4 (3.1)	22.4 (3.0)
**Physical activity (METs-h/week) (%)**	15.8 (18.0)	18.5 (19.0)	22.6 (21.5)
**Years at university (%)**	4.8 (1.3)	4.8 (1.3)	4.9 (1.4)
**Height (cm) (%)**	164 (6)	164 (6)	163 (6)
**Breastfeeding (months) (%)**	1.9 (4.6)	2.4 (4.8)	2.7 (5.2)
**Hormone replacement therapy (%)** ^**a**^			
No	61.1	65.3	61.7
Yes	38.9	34.8	38.3
**Time of hormone replacement therapy (years)** ^**a**^	1.3 (2.3)	1.4 (2.6)	1.4 (2.5)
**Family history of diabetes (%)**			
No	88.5	84.9	81.9
Yes	11.5	15.1	18.2
**Smoking (%)**			
Never	53.6	52.0	48.9
Current smoker	26.1	22.6	19.1
Former smoker	20.3	25.4	32.0
**Lifetime tobacco exposure (pack-years) (%)**	3.4 (6.1)	4.3 (7.1)	5.0 (8.2)
**Television watching (hours/day)**	1.7 (1.3)	1.6 (1.2)	1.5 (1.2)
**Family history of breast cancer (%)**			
None	89.9	89.1	89.1
Before 45 years	1.8	1.9	1.9
After 45 years	8.4	9.1	9.1
**Age at menarche (%)**			
<=9 years	1.0	1.2	1.3
10–11 years	17.5	19.2	21.0
12–13 years	54.5	56.1	53.5
≥ 14 years	27.0	23.5	24.2
**Menopausal status at recruitment (%)**			
Premenopausal (%)	97.2	92.5	86.6
Postmenopausal (%)	2.8	7.5	13.4
**Age at menopause** ^**a**^			
Postmenopausal < 50 years (%)	41.7	40.4	41.5
Postmenopausal ≥ 50 years (%)	58.3	59.6	58.5
**Obstetric history (%)**			
Age < 25 years and nulliparous	24.9	16.8	10.9
Age ≥ 25 years and nulliparous	47.4	49.0	51.6
First pregnancy before the age of 30 years	15.9	19.7	22.0
First pregnancy being 30 years old or older	11.9	14.5	15.5
**Oral contraceptives**			
No	97.3	97.5	98.0
Yes	2.7	2.5	2.1
**Dietary intakes**			
**Alcohol intake (g/d)**	4.1 (5.9)	4.1 (5.9)	3.9 (5.7)
**Total energy intake (kcal/d)**	2464 (534)	2218 (577)	2193 (568)
Carbohydrate (% of E)	41.2 (7.1)	43.2 (6.9)	45.7 (7.3)
Protein (% of E)	18.0 (3.2)	18.5 (3.4)	18.8 (3.4)
Total fat (% of E)	39.6 (6.0)	36.9 (6.3)	34.2 (6.6)
MUFAs (% of E)	16.8 (3.5)	16.1 (4.0)	15.1 (4.0)
SFAs (% of E)	5.3 (1.6)	5.1 (1.6)	5.0 (1.5)
PUFAs (% of E)	14.1 (3.0)	12.4 (2.7)	10.6 (2.8)

a: only among postmenopausal women

To assess the relation between cumulative average adherence to DDS and the subsequent risk of breast cancer, we used Cox regression models with age as underlying time variable, stratified by age (decades) and recruitment period (three periods) and included confirmed incident breast cancer as the main outcome in the overall sample (*n* = 10,810 women) and by menopausal status. Reference category was the lowest tertile. Follow-up was considered for each participant from the baseline questionnaire return date until breast cancer diagnosis for the cases and death or end of follow-up for the non-cases. For all analyses and to control for potential confounders, we used multivariable adjusted models adjusting for the aforementioned potential confounders.

Thereafter, we further applied stratified analysis among pre- and postmenopausal women and their risk of breast cancer. Information on age at menopause was collected in the baseline questionnaire and after 18 years of follow-up. For the assessment of premenopausal breast cancer as the outcome, women who reported being menopausal before baseline assessment were excluded, and we censored follow-up time at the age of 52 years or at the self-reported age of menopause, whichever occurred first. When assessing postmenopausal breast cancer, women were considered at risk only after having turned 52 years old or after their self-reported age of menopause, whichever occurred last. For initially premenopausal women who turned postmenopausal during follow-up, we calculated time since recruitment as the difference between the self-reported date of menopause and the date of completion of the baseline assessment [30]. Among premenopausal women, we did not adjust for age at menopause and use of hormone replacement therapy. Among postmenopausal women, we also adjusted for the time between recruitment and menopause, but not for oral contraceptive use. Analyses for premenopausal breast cancer were right-censored when women turned 52 years old and analyses for postmenopausal breast cancer were left-censored at the age of 52 years.

Distinction between pre and postmenopausal breast cancer is crucial due to their distinct behaviors and risk factors. Premenopausal and postmenopausal breast cancers are associated with different risk factors, such as reproductive history, hormone exposure, and genetic predisposition. Moreover, this classification allows for a more precise identification of risk factors and the development of tailored prevention and treatment strategies.

We also conducted tests of linear trend for the evaluation of dose–response relationships, assigning its tertile-specific median to each category of the baseline adherence to DDS and using the resulting variable as continuous in the abovementioned models. We also conducted a test to evaluate a possible interaction between BMI (< 25 kg/m^2^ and ≥25 kg/m^2^) and DDS score in the overall sample after 10-years of follow-up.

Analyses were carried out using Stata version 16.0 (Stata Corporation). All *p* values were two-tailed and a *p* value < 0.05 was deemed as statistically significant.

## Results

For the analysis, 10,810 women were included, with a mean follow-up of 12.5 years. Baseline characteristics of participants are described in Table 1 according to tertiles of adherence to DDS. Women with the higher adherence to the DDS were more physically active, had a higher prevalence of diabetes and a higher prevalence of family history of diabetes and were more likely to be former smokers. Regarding dietary intake, women in this category had a lower energy intake and showed a higher intake of carbohydrates and a lower intake of total fat.

### DDS and overall breast cancer

During 122,020 person-years of follow-up, 147 invasive breast cancer cases were confirmed. Compared to women with the lowest adherence to the DDS, women with a moderate adherence to DDS (second tertile (T2), median range of 33 points) showed a significantly lower risk of breast cancer (HR_T2 vs. T1_= 0.55; 95% CI: 0.36–0.82) in the fully adjusted model (Table 2).

**Table 2:** Hazard ratio (HR) and confidence intervals 95% (CI 95%) of breast cancer according to tertiles of the diabetes risk score in the overall sample of 10,810 women from the SUN cohort (1999–2019)

	Dietary-Based Diabetes Risk Score			
	Tertile 1 (T1)	Tertile 2 (T2)	Tertile 3 (T3)	*p* for trend
N	3,805	3,781	3,224	
Median (range)	27 (13–30)	33 (31–36)	40 (37–54)	
Incident cases	59	37	51	
Person-years of follow-up	44,534	42,633	34,852	
Incidence rate/10,000 person-years	13.2	8.7	14.6	
Age-adjusted	1 (Ref.)	0.52 (0.34–0.78)	0.75 (0.51–1.10)	0.17
Multivariable adjusted	1 (Ref.)	0.55 (0.36–0.82)	0.78 (0.52–1.17)	0.31

All Cox models were stratified for age (decades) and recruitment period. The multivariable adjusted model was adjusted for height, years at university, family history of breast cancer (none, after 45 years, or before 45 years), smoking status (never smoker, former smoker, current smoker), lifetime tobacco exposure (pack-years), physical activity (METs-h/week), TV watching (h/day), alcohol intake (g/day, continuous), BMI (< 25, 25–30, ≥ 30 kg/m^2^), age of menarche (< 10 years, 10–11 years, 12–13 years, ≥ 14 years), age at menopause (< 50 years, ≥ 50 years), history of pregnancy (age < 25 years and nulliparous, age ≥ 25 years and nulliparous, first pregnancy before the age of 30 years, first pregnancy being 30 years old or older), months of breastfeeding (continuous), use of hormone replacement therapy (yes/no), energy intake (kcal/day), prevalence of diabetes, family history of diabetes, and use of oral contraceptives (yes/no). Cumulative averages for all dietary variables were used

**Fig. 2:**
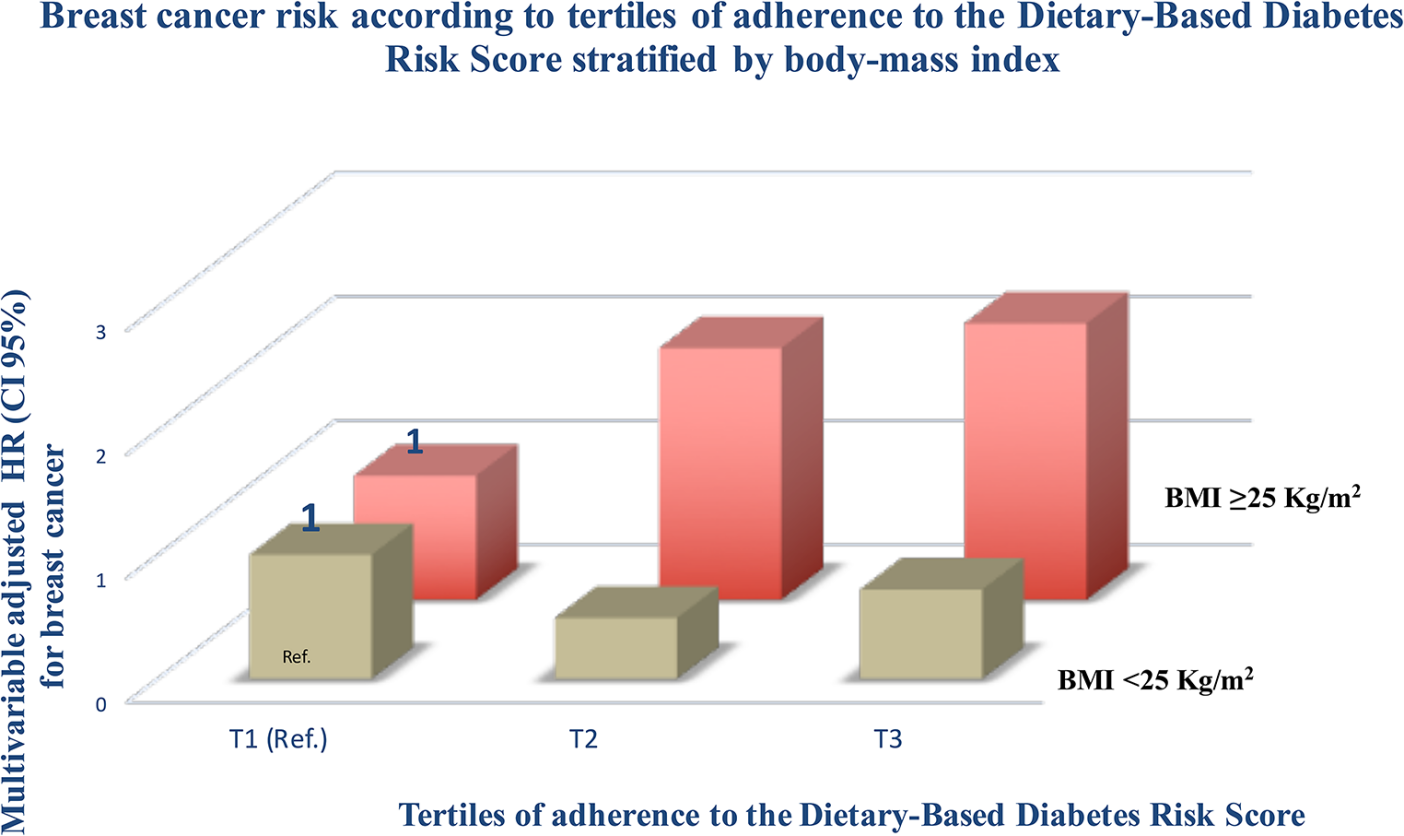
Hazard ratio (95% CI) of breast cancer according to tertiles of adherence to the Dietary-Based Diabetes Risk Score (DDS) and BMI strata (normal weight and overweight/obesity). Adjusted for height, years at university, family history of breast cancer (none, after 45 years, or before 45 years), smoking status (never smoker, former smoker, current smoker), lifetime tobacco exposure (pack-years), physical activity (METs-h/week), TV watching (h/day), alcohol intake (g/day, continuous), BMI (< 25, 25–30, ≥ 30 kg/m2), age at menarche (< 10 years, 10–11 years, 12–13 years, ≥ 14 years), age at menopause (< 50 years, ≥ 50 years), history of pregnancy (age < 25 years and nulliparous, age ≥ 25 years and nulliparous, first pregnancy before the age of 30 years, first pregnancy being 30 years old or older), months of breastfeeding (continuous), use of hormone replacement therapy (yes/no), energy intake (kcal/day), prevalence of diabetes, family history of diabetes, and use of oral contraceptives (yes/no). Cumulative averages over follow-up were used for all dietary variables

Moreover, results from the interaction analysis between DDS score and BMI were statistically significant with different estimates for normal weight women (BMI < 25 kg/m^2^) (HR_T2 vs. T1_= 0.49; 95% CI: 0.31–0.78) and (HR_T3 vs. T1_= 0.72; 95% CI: 0.46–1.11) in the fully adjusted model (p_interaction_: 0.029), whereas these estimates were (HR_T2 vs. T1_= 2.03; 95% CI: 0.40–10.20) and (HR_T3 vs. T1_= 2.23; 95% CI: 0.45–11.03) among overweight/obese women (Fig. 2).

### DDS and premenopausal breast cancer

When we divided participants according to menopausal status, among premenopausal women, again women with a moderate adherence to DDS (second tertile, median range of 33 points) showed a lower premenopausal breast cancer risk in the fully adjusted model compared to those women with the lowest adherence to DDS (HR_T2_= 0.26; 95% CI: 0.13–0.53) (Table 3) considering updated cumulative information after 10-years of follow-up.

**Table 3:** Hazard ratio (HR) and confidence intervals 95% (CI 95%) of premenopausal breast cancer according to tertiles of the Dietary-Based Diabetes Risk Score in the SUN cohort (1999–2019)

	Dietary-Based Diabetes Risk Score			
	Tertile 1	Tertile 2	Tertile 3	*p* for trend
N	3,672	2,927	3,289	
Median (range)	27 (13–30)	33 (31–36)	40 (37–54)	
Incident cases	42	9	34	
Person-years of follow-up	38,876	28,689	29,412	
Incidence rate/10,000 person-years	10.8	3.1	11.6	
Age-adjusted	1 (Ref.)	0.25 (0.12–0.51)	0.85 (0.54–1.34)	0.372
Multivariable adjusted	1 (Ref.)	0.26 (0.13–0.53)	0.92 (0.57–1.49)	0.603

Results from Cox regression models. All Cox models were stratified for age (decades) and recruitment period. The multivariable adjusted model was adjusted for height, years at university, family history of breast cancer (none, after 45 years, or before 45 years), smoking status (never smoker, former smoker, current smoker), lifetime tobacco exposure (pack-years), physical activity (METs-h/week), TV watching (h/day), alcohol intake (g/day, continuous), BMI (< 25, 25–30, ≥ 30 kg/m^2^), age of menarche (< 10 years, 10–11 years, 12–13 years, ≥ 14 years), history of pregnancy (age < 25 years and nulliparous, age ≥ 25 years and nulliparous, first pregnancy before the age of 30 years, first pregnancy being 30 years old or older), months of breastfeeding (continuous), energy intake (kcal/day), prevalence of diabetes, family history of diabetes and use of oral contraceptives (yes/no). Cumulative averages were used for all dietary variables

### DDS and postmenopausal breast cancer

On the other hand, we did not find any significant association between tertiles of adherence to the DDS and postmenopausal breast cancer (Table 4).

**Table 4:** Hazard ratio (HR) and confidence intervals 95% (CI 95%) of postmenopausal breast cancer according to tertiles of the Dietary-Based Diabetes Risk Score in the SUN cohort (1999–2019)

	Dietary-Based Diabetes Risk Score			
	Tertile 1	Tertile 2	Tertile 3	*p* for trend
N	776	959	809	
Median (range)	29 (14–32)	35 (33–38)	42 (39–54)	
Incident cases	19	24	14	
Person-years of follow-up	5764	8471	7740	
Incidence rate/10,000 person-years	33.0	28.3	18.1	
Age-adjusted	1 (Ref.)	0.90 (0.49–1.65)	0.58 (0.29–1.16)	0.105
Multivariable adjusted	1 (Ref.)	1.05 (0.56–1.96)	0.67 (0.33–1.34)	0.186

All Cox models were stratified for age (decades) and recruitment period. Multivariable adjusted model was adjusted for height, years at university, family history of breast cancer (none, after 45 years, or before 45 years), smoking status (never smoker, former smoker, current smoker), lifetime tobacco exposure (pack-years), physical activity (METs-h/week), TV watching (h/day), alcohol intake (g/day, continuous), BMI (< 25, 25–30, ≥ 30 kg/m^2^), age at menarche (< 10 years, 10–11 years, 12–13 years, ≥ 14 years), age at menopause (< 50 years, ≥ 50 years), history of pregnancy (age < 25 years and nulliparous, age ≥ 25 years and nulliparous, first pregnancy before the age of 30 years, first pregnancy being 30 years old or older), months of breastfeeding (continuous), use of hormone replacement therapy (yes/no), energy intake (kcal/day), prevalence of diabetes, family history of diabetes, and time between recruitment and menopause. Cumulative averages were used for all dietary variables

## Discussion

In the SUN Project, women with a moderate adherence to the DDS showed a lower risk of breast cancer after a median follow-up time of 12.5 years. This association was somewhat stronger among women with a BMI < 25 kg/m^2^. This result was also observed for premenopausal breast cancer, whereas this association was no longer observed for postmenopausal breast cancer.

One possible explanation for the observed difference between premenopausal and postmenopausal women in the association between DDS and breast cancer risk could be related to the metabolic differences between pre- and postmenopausal breast cancer. Among premenopausal women, breast cancer is often associated with factors such as high insulin levels and insulin resistance. An antidiabetic diet, which typically focuses on reducing sugar and refined carbohydrates, may help to reduce insulin levels and improve insulin sensitivity, thus lowering the risk of breast cancer. On the other hand, among postmenopausal women, breast cancer is more commonly associated with factors such as estrogen levels and estrogen receptor-positive tumors. An antidiabetic diet, which often includes higher consumption of fruits, vegetables, and whole grains, may lead to higher estrogen levels due to the phytoestrogens present in these foods, potentially increasing the risk of breast cancer in postmenopausal women. Therefore, our hypothesis is that the protective effect of an antidiabetic diet against breast cancer observed in premenopausal women may be outweighed by the potential risk associated with increased estrogen levels in postmenopausal women, leading to the lack of significant association between DDS and breast cancer risk in this group.

Several recently published studies have explored the association between a diabetes risk reduction diet (DRRD) score and some other types of cancer [22, 31, 32]. We identified some differences between DDS and DRRD score [19, 22]. Firstly, the DDS score considers fiber from the whole diet, whereas the DRRD score only considers fiber from cereals. Secondly, the DDS score considers only PUFA intake, whereas the DRRD score considers the ratio of PUFA to saturated fatty acids (P: S ratio). In addition, and in terms of the type of fat, the latter adds the information on trans fats in the diet and the glycemic index. Finally, only the DDS takes into consideration the group of vegetables, fruits, low fat dairy and whole grain cereals. Notwithstanding, our results were partially consistent with another study in which a higher adherence to the DRRD score [33] showed a lower risk of incident invasive breast cancer with an adherence score similar to ours [34]. In a case-control study, higher adherence to the DRRD score was inversely associated with pancreatic cancer [31]. In contrast to our study, the median age of the study population in that study was higher (63 years). Similarly, in another case-control study, higher adherence to DRRD score was associated with 27% lower odds of endometrial cancer, even after exclusion of women with diabetes [13]. Moreover, in the Nurses’ Health Study and Health Professionals Follow-up Study [22], the DRRD was inversely associated with the risk of developing hepatocellular carcinoma. To the best of our knowledge, only a hospital-based case-control study [35] has assessed the relationship between the DDS and colorectal cancer and colorectal adenoma. Concretely, they found an inverse relationship between DDS and the odds of colorectal cancer and colorectal adenoma.

Overall, an inverse association between a moderate adherence to DDS score and breast cancer risk could be attributed to higher consumption of the healthier food groups such as vegetables, fruits, and dairy products. The recommended dietary pattern for breast cancer prevention is made up predominantly of vegetables, fruits, whole grains, beans and other plant foods, with limited– if any– red meat, sugary foods and alcohol [36].

There are some biological mechanisms that could help us understand the observed associations. The DDS reflects an antidiabetic diet that may have a potential preventive effect on breast cancer through the reduction of underlying mechanisms which are common to diabetes development. As such, a higher adherence to the DDS may help to prevent metabolic dysregulations as a result of hyperinsulinemia, hyperglycemia and chronic inflammation [37]. In fact, the DDS could be considered as a diverse diet with anti-inflammatory and anti-insulinemic potential. It includes a combination of food groups that contain different food micronutrients -carotenoids, vitamin C, phytosterols-, which are widely known to have antioxidants and anti-carcinogenic properties [19].

Our finding of stronger inverse DDS associations with premenopausal breast cancer –for which factors related to adiposity/hyperinsulinemia may be most important– should be highlighted considering the importance of a diet with an anti-diabetic profile may have on premenopausal women. Numerous studies have shown that adiposity, characterized by excess body fat, is associated with hyperinsulinemia and increased levels of insulin-like growth factors (IGFs). These hormonal changes can promote cell proliferation and inhibit apoptosis in breast tissue, thereby contributing to the development of breast cancer, particularly in premenopausal women [38–40, 28, 35]. For example, a meta-analysis by Harvie et al. found that higher levels of insulin and IGF-1 were associated with an increased risk of premenopausal breast cancer [39]. Similarly, a large prospective cohort study by Eliassen et al. reported that higher BMI during early adulthood was associated with an increased risk of premenopausal breast cancer, with insulin and IGF-1 mediating a significant proportion of this association [40]. Therefore, in the context of our study, which found an inverse association between DDS score and breast cancer risk, it is plausible that the protective effect of a diverse diet against breast cancer may be more pronounced in premenopausal women, where factors related to adiposity and hyperinsulinemia are most influential.

In our study, we observed a significant interaction with BMI so that the inverse association was only apparent for women with BMI < 25 kg/m^2^, but not among overweight or obese women. It is important to highlight that a similar interaction was observed in the Nurses’ Health Study, in which the inverse association between adherence to the DRRD and breast cancer risk was strongest among women with BMI < 25 kg/m^2^ [34]. Since one of the most important risk factors for T2D and breast cancer is BMI, our results suggest that the relationship between an antidiabetic diet and the future risk of breast cancer may vary depending on BMI. The DDS seemed to significantly reduce the risk among women with BMI < 25 kg/m^2^, but not among women with BMI > = 25 kg/m^2^. In the latter group, the association yielded no significant associations. We hypothesize that the mechanisms through which the DDS could reduce the risk of breast cancer among women with BMI < 25 kg/m^2^ might be already activated among women with a higher BMI, so that the DDS could no longer exert its beneficial effects. A possible explanation would be that among women without overweight or obesity (BMI < 25 kg/m^2^), the potentially protective effect of higher adherence to the DDS score would be associated, first, with a healthier dietary profile and, in the long-term, with a lower proinflammatory state. Thus, the combination of an appropriate antidiabetic diet and maintenance of a healthy BMI could substantially lower the risk of breast cancer through the prevention of insulin-resistance. Both obesity and T2D are associated with insulin resistance [41]. Hitherto, prospective observational studies examining insulin levels and breast cancer incidence have shown direct associations [42–44]. Therefore, both weight control and an antidiabetic dietary pattern, which prevent insulin resistance, would be promising strategies to reduce breast cancer risk.

The current study has some strengths, namely its prospective design, updated dietary measurements, long-term follow-up, collection of dietary information with a previously validated FFQ [25, 26] and confirmation of breast cancer cases. However, we acknowledge some limitations, including the lower number of confirmed incident breast cancer cases that provide somewhat unstable risk estimates and the younger age of our cohort which might have the limited power to further explore definitive associations for postmenopausal women. Finally, residual confounding cannot be completely ruled out despite the adjustment for a wide array of potential confounders.

## Conclusion

In conclusion, we found that a moderate adherence to a T2D prevention diet assessed by the DDS was inversely associated with the risk of developing breast cancer. This observed association was stronger among women with a lower BMI. Furthermore, higher adherence to the antidiabetic diet may be a good option especially for women with BMI < 25 kg/m^2^. More studies, especially in high-risk populations, are needed.

## Electronic supplementary material

Supplementary material
