# Supplementary material for: Dietary-Based Diabetes Risk Score and breast cancer: a prospective evaluation in the SUN project
Source: J Physiol Biochem. 2024 Sep 5;81(2):459–69. doi: 10.1007/s13105-024-01036-9 (PMC12279892; doi:10.1007/s13105-024-01036-9)
Supplement: Supplementary file 1 — Supplementary material [file 13105_2024_1036_MOESM1_ESM.docx]

**Supplemental eTable 1**: Scoring criteria for the Dietary-Based Diabetes Risk Score (DDS) in the SUN cohort, 1999–2019.

| **COMPONENT** | **INCLUDED FOODS** |
| --- | --- |
| **Decreased risk** | |
| Vegetables | Carrot, pumpkin, Swiss chard, cabbage, cauliflower, broccoli, lettuce, chicory, escarole, tomatoes, green beans, eggplant, zucchini, cucumber, peppers, asparagus, spinach, other fresh vegetables |
| Fruits | Citrus, grapes, banana, apple, pear, strawberry, peach, apricot, nectarine, cherries, plums, figs, melon, watermelon, grapes, mango, papaya, kiwi |
| Total dietary fiber | g/day |
| Whole-grain cereals | Whole-grain bread |
| Nuts | Almonds, peanuts, hazelnuts, walnuts |
| Coffee | Cups (50 mL) of coffee consumed |
| Polyunsaturated fatty acids (PUFA) | g/d |
| Low-fat dairy products | Skim or low-fat milk and cheese |
| **Increased risk** | |
| Red meat | Beef, veal, pork, lamb, liver, other viscera |
| Processed meat | Serrano ham, cooked ham, spicy pork sausage, salami, mortadella, foie gras, black pudding, bacon, other cured or smoked meats, hamburger, hot dog. |
| Sugar-sweetened beverages (SSBs) and fruit juices | Sugar-sweetened beverages, bottled fruit juice |

Consumption (g/d) of eight nutritional exposures which have shown associations with a decreased incidence of T2D (vegetables, fruit, fiber, whole cereals, nuts, coffee, PUFA, low-fat dairy), and three food groups which have shown associations with an increased incidence of T2D (red meat, processed meat, and sugar-sweetened beverages [SSB]) were considered. All the components were adjusted by using the residuals method. The overall Dietary-Based Diabetes-Risk Score (DDS) was built by summing both components with a range from 11-55 points.

| **Supplemental eTable 2.** Age-adjusted baseline characteristics of participants according to categories of the Diabetes Risk Score: The Seguimiento Universidad de Navarra (SUN) cohort: 1999-2019. | | | |
| --- | --- | --- | --- |
| **Tertiles of DDS score** | | | |
| **Variable** | **T1** | **T2** | **T3** |
| **N** | 3,805 | 3,781 | 3,224 |
| **Median DDS score (range)** | 27 (13-30) | 33 (31-36) | 40 (37-54) |
| **Age (years)** | 35 (11) | 35 (11) | 35 (11) |
| **Body mass index (kg/m^2^) (%)** | 22.1 (3.3) | 22.4 (3.1) | 22.1 (2.9) |
| **Physical activity (METs-h/week) (%)** | 15.6 (17.6) | 18.6 (19.1) | 23.1 (22.2) |
| **Years at university (%)** | 4.8 (1.3) | 4.8 (1.3) | 4.8 (1.4) |
| **Height (cm)(%)** | 164 (6) | 164 (6) | 164 (6) |
| **Breastfeeding (months) (%)** | 2.5 (5.3) | 2.3 (4.8) | 2.1 (4.7) |
| **Hormone replacement therapy (%) ^a^** |  |  |  |
| No | 62.3 | 65.8 | 61.3 |
| Yes | 37.7 | 34.2 | 38.7 |
| **Time of hormone replacement therapy (years) ^a^** | 1.3 (2.3) | 1.4 (2.5) | 1.4 (2.5) |
| **Family history of diabetes (%)** |  |  |  |
| No | 87.5 | 85.1 | 83.5 |
| Yes | 12.5 | 14.9 | 16.5 |
| **Smoking (%)** |  |  |  |
| Never | 51.5 | 52.4 | 51.7 |
| Current smoker | 25.3 | 22.6 | 19.9 |
| Former smoker | 23.2 | 25.0 | 28.4 |
| **Lifetime tobacco exposure (pack-years) (%)** | 4.2 (7.1) | 4.2 (7) | 4.2 (7.2) |
| **Hours/day television watching (%)** | 1.7 (1.3) | 1.6 (1.2) | 1.5 (1.2) |
| **Family history of breast cancer (%)** |  |  |  |
| None | 89.5 | 89.0 | 89.4 |
| Before 45 years | 2.0 | 1.9 | 1.8 |
| After 45 years | 8.5 | 9.1 | 8.9 |
| **Age at menarche (%)** |  |  |  |
| <= 9 years | 1.1 | 1.2 | 1.4 |
| 10-11 years | 17.7 | 19.1 | 20.4 |
| 12-13 years | 54.0 | 56.2 | 53.7 |
| ≥14 years | 27.2 | 23.5 | 24.6 |
| **Menopausal status at recruitment (%)** |  |  |  |
| Premenopausal (%) | 93.3 | 92.4 | 92.1 |
| Postmenopausal (%) | 6.7 | 7.6 | 7.9 |
| **Age at menopause ^a^** |  |  |  |
| Postmenopausal < 50 years (%) | 39.1 | 40.2 | 42.4 |
| Postmenopausal ≥ 50 years (%) | 60.9 | 59.8 | 57.6 |
| **Obstetric history (%)** |  |  |  |
| Age <25 years and nulliparous | 18.0 | 18.1 | 17.1 |
| Age ≥25 years and nulliparous | 46.3 | 48.6 | 53.3 |
| First pregnancy before de age of 30 years | 21.0 | 19.2 | 16.9 |
| First pregnancy being 30 years old or older | 14.7 | 14.1 | 12.7 |
| **Oral contraceptives** |  |  |  |
| No | 97.8 | 97.5 | 97.4 |
| Yes | 2.2 | 2.5 | 2.7 |
| **Dietary intakes** |  |  |  |
| **Alcohol intake (g/d)** | 4.2 (6.3) | 4.1 (5.9) | 3.7 (5.4) |
| **Total energy intake (kcal/d)** | 2461 (541) | 2218 (577) | 2197 (568) |
| Carbohydrate (% of E) | 41.2 (7.3) | 43.3 (7.0) | 45.7 (7.2) |
| Protein (% of E) | 18.1 (3.4) | 18.5 (3.4) | 18.8 (3.4) |
| Total fat (% of E) | 39.5 (6.1) | 36.9 (6.3) | 34.3 (6.5) |
| MUFAs (% of E) | 16.8 (3.6) | 16.0 (4.0) | 15.1 (3.9) |
| SFAs (% of E) | 5.3 (1.6) | 5.1 (1.6) | 5.0 (1.6) |
| PUFAs (% of E) | 14.1 (3.0) | 12.4 (2.7) | 10.6 (2.7) |

*Adjusted for age with inverse probability weighting

a: Only for postmenopausal women
